# Galactosylated Chitosan-Functionalized Mesoporous Silica Nanoparticle Loading by Calcium Leucovorin for Colon Cancer Cell-Targeted Drug Delivery

**DOI:** 10.3390/molecules23123082

**Published:** 2018-11-26

**Authors:** Wei Liu, Fan Wang, Yongchao Zhu, Xue Li, Xiaojing Liu, Jingjing Pang, Weisan Pan

**Affiliations:** 1 Department of Pharmaceutics, School of Pharmacy, Shenyang Pharmaceutical University, 103 Wenhua Road, Shenyang 110016, China; liuweiyxy@zzu.edu.cn; 2 Department of Pharmaceutics, School of Pharmacy, Zhengzhou University, 100 Science Avenue, Zhengzhou 450001, China; 18739918527@163.com (F.W.); zychenu@163.com (Y.Z.); 18992836789@189.cn (X.L.); 15890767550@163.com (X.L.); 18369609056@163.com (J.P.)

**Keywords:** calcium leucovorin, MSN–COOH, galactosylated chitosan (GC), LV@MSN–COOH/GC, galectin receptor, SW620 cells

## Abstract

Targeted drug delivery to colon cancer cells can significantly improve the efficiency of treatment. We firstly synthesized carboxyl-modified mesoporous silica nanoparticles (MSN–COOH) via two-step synthesis, and then developed calcium leucovorin (LV)-loaded carboxyl-modified mesoporous silica nanoparticles based on galactosylated chitosan (GC), which are galectin receptor-mediated materials for colon-specific drug delivery systems. Both unmodified and functionalized nanoparticles were characterized by scanning electron microscopy (SEM), transmission electron microscope (TEM), X-ray diffraction (XRD), Fourier transform infrared (FT-IR), nitrogen sorption, and dynamic light scattering (DLS). Drug release properties and drug loading capacity were determined by ultraviolet spectrophotometry (UV). LV@MSN–COOH/GC had a high LV loading and a drug loading of 18.07%. In vitro, its release, mainly by diffusion, was sustained release. Cell experiments showed that in SW620 cells with the galectin receptor, the LV@MSN–COOH/GC metabolized into methyl tetrahydrofolic acid (MTHF) and 5-fluorouracil (5-FU)@MSN–NH_2_/GC metabolized into FdUMP in vivo. MTHF and 5-fluoro-2′-deoxyuridine 5′-monophosphate (FdUMP) had combined inhibition and significantly downregulated the expression of thymidylate synthase (TS). Fluorescence microscopy and flow cytometry experiments show that MSN–COOH/GC has tumor cell targeting, which specifically recognizes and binds to the galectin receptor in tumor cells. The results show that the nano-dosing system based on GC can increase the concentrations of LV and 5-FU tumor cells and enhance their combined effect against colon cancer.

## 1. Introduction

Colorectal cancer (CRC) is the fourth most frequent cause of cancer-related mortality in China [1]. Classical clinical treatments for this condition include surgery, radiotherapy, and most commonly, chemotherapy. Among the different chemotherapeutics used to treat colorectal cancer, 5-fluorouracil (5-FU), a pyrimidine analog that acts as a thymidylate synthase (TS) inhibitor, is a first-line drug due to its low price and effective anticancer activity [2,3].

Calcium leucovorin (LV) is a folate-reduced formylated derivative that is metabolized to methyl tetrahydrofolic acid (MTHF) in tumor cells. As a rate-limiting enzyme for DNA synthesis, TS can form a triple complex with 5-fluoro-2′-deoxyuridine 5′-monophosphate (FdUMP) produced by 5-FU in the presence of folic acid cofactor LV, which competes with the intermediates of the uridine synthesis thymidylate process. The triple complex inhibits the formation of deoxythymidylate, thereby blocking the synthesis of thymidylate, ultimately hindering the biosynthesis of DNA, which inhibits the growth and proliferation of tumor cells [4,5,6,7]. Due to its unstable oral bioavailability and rapid metabolism by dihydropyrimidine dehydrogenase after oral administration, calcium leucovorin (LV) is usually administered intravenously or via bolus injection, and the combination of 5-FU and LV has become the first-line treatment for colon cancer chemotherapy worldwide [2,3]. It can significantly improve overall survival compared with 5-FU treatment alone [8,9]. In patients with metastatic colorectal cancer treated with FU/LV, it was shown that approximately 20% of tumors were reduced by 50% or more, and the average survival increased from ~6 months to ~12 months [10].

However, continuous intravenous injection of 5-FU can cause serious systemic adverse reactions, such as diarrhea, hand and foot syndrome, mucositis/stomatitis, neutropenia, anemia, nausea/vomiting, cardiotoxicity, increased myocardial enzymes, discoloration along the vein through which medication is given, thrombocytopenia, and myelosuppression [11,12]. There is much literature related to clinical and molecular biological studies of the 5-FU/LV inhibition of colon cancer, but few studies investigated drug delivery systems, with most of these being conducted in vitro [13,14,15]. Li and others prepared nanoparticles containing both Fu and LV using an ion cross-linking method with chitosan as a carrier. The nanoformulation prepared by Li et al. was subjected to an in vitro release study in phosphate-buffered saline (PBS) and their study lacked research on cell targeting of the formulation and did not show that the combination of LV and 5-FU enhanced the anti-tumor activity of 5-FU [14].

Among the various nanocarriers, mesoporous silica nanoparticles (MSNs) garnered particular attention and were developed into ideal hosts for controlled 5-FU drug delivery [16,17,18,19,20]. Compared with other therapeutic carriers, they offer several advantages including low cytotoxicity, a high surface area, low mass density, a tunable size, and a pore diameter that allows fine control of the drug load and release kinetics, guest molecules with a high adsorption capacity, and high chemical and mechanical stability [21,22,23,24]. More importantly, due to the abundant silanol groups on the surface, MSNs are easily modified with functional groups to allow better control over drug loading and release [25]. Various organic groups of functionalized MSNs were developed to control 5-FU release [18,23,26,27,28,29]. However, thus far, MSN materials are not reported in the literature as carriers of LV. In our study, a carboxylated MSN (MSN–COOH) material is used as a carrier for LV.

Galactosylated chitosan (GC), a derivative of chitosan (CS) that is hydrosoluble at neutral pH, is synthesized by covalently binding d-galactose units to CS through *O*-1,6 glycosidic linkages [30,31]. A previous study showed that, compared with CS, GC has better hydrosolubility and mucoadhesiveness. More importantly, GC has better cell compatibility and lower toxicity. [32]. Although galactosylated chitosan was proven to significantly enhance the targeting ability of hepatocytes [12,33,34,35], studies on their colon-targeting are limited. Several studies showed that galactosylated chitosan can deliver drugs to activated colonic macrophages via galactose receptor-mediated endocytosis [36,37]. In addition, many studies showed that galectin is overexpressed in colorectal cancer and plays a key role in regulating its development and metastasis [38,39,40]. In addition, galectin has a high affinity for natural small sugars such as galactose and lactose [38]. According to what we know, no one studied whether GC can be used as a vector for targeting colorectal cancer.

**Figure 1:**
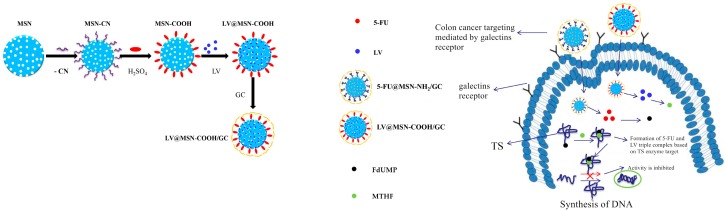
Synthesis of calcium leucovorin (LV)-loaded carboxyl-modified mesoporous silica nanoparticles based on galactosylated chitosan (LV@MSN–COOH/GC) and its mechanism of drug release in SW620 cells.

In this part of the study, it is hoped that a colon-targeted drug delivery system of LV can be successfully constructed to target colon tumor cells, to increase its uptake by colon tumor cells, and to better bind to 5-FU metabolites and TS to enhance the inhibition of tumor cells. Binding enhances the inhibition of tumor cells. MSN–COOH was prepared via a two-step reaction, coated with galactosylated chitosan, and used as a carrier material to support LV, focusing on the colon-targeting of LV mesoporous silica. The process is indicated in Figure 1, showing the in vitro targeting effect of the drug system on the human colon cancer cell line SW620 and its combined effect with the 5-FU formulation.

## 2. Materials and Methods

### 2.1. Materials

Folinic acid calcium salt hydrate (LV) was purchased from Dalian Meilun Biological Technology Co., Ltd. (Dalian, China). Tetraethylorthosilicate (TEOS) was obtained from Sinopharm Chemical Reagents Co., Ltd. (Shanghai, China). Hexadecyl trimethyl ammonium bromide (CTAB) was purchased from Beijing Dingguo Changsheng Biological Technology Co., Ltd. (Beijing, China), while *N*,*N*-dimethylformamide was purchased from Tianjin Tianxin Fine Chemical Development Center (China). All of these regents were of analytical grade. *N*-Hydroxysuccinimide (NHS) and 1-(3-dimethylaminopropyl)-3-ethylcarbodiimide hydrochloride (EDC) were purchased from Shanghai Aladdin Bio-Chem Technology Co., Ltd. (China). Dulbecco’s modified Eagle medium (DMEM) was purchased from Beijing Heikelong Technology Co., Ltd. (Beijing, China). Fetal bovine serum (FBS) was purchased from Biological Industries (Kibbutz Beit Haemek, Israel). Penicillin and streptomycin were purchased from Genom biological medicine technology Co., Ltd. (Hangzhou, China). Chitosan (molecular weight, 40–60 kDa; deacetylation degree, 91.3%) was purchased from Qingdao Yunzhou Biological Science and Technology Co., Ltd. (Qingdao, China). All chemicals mentioned above were used without further purification. Deionized water was used throughout the experiments.

### 2.2. Preparation of MSN–COOH–GC and MSN–COOH/GC

#### 2.2.1. Synthesis of Carboxyl Functionalized Mesoporous Silica Nanoparticles (MSN–COOH)

MSN–COOH was synthesized via a two-step synthesis. While stirring, 1.2 g of CTAB was dissolved in a mixed solution of 180 mL of ultrapure water and 5.5 mL of ammonia water (25%), and stirred vigorously at 60 °C for 30 min. Subsequently, 2.0 mL of TEOS and 0.4 mL of 2-cyanoethyltriethoxysilane were added quickly, and the reaction continued for 2 h. It was then aged at 60 °C for 24 h and centrifuged at 20,000 rpm for 20 min to obtain a crude product. It was separately dispersed twice with deionized water and ethanol, and dried at 50 °C. The product was dried and added to 30 mL of 9 mol/L sulfuric acid, and reacted in an oil bath at 100 °C for 18 h. After the reaction was complete, it was centrifuged at 20,000 rpm for 20 min, and the product was further treated with 9% hydrochloric acid in ethanol at 65 °C for 24 h. Subsequently, it was centrifuged at 20,000 rpm for 20 min and dried at 50 °C overnight to obtain the carboxyl-modified MSN (MSN–COOH) [41,42,43,44].

#### 2.2.2. Synthesis of Galactosylated Chitosan (GC)

Chitosan (2.8 g) and d-galactose (6.0 g) were added to anhydrous tetrahydrofuran (THF; 600 mL), and boron trifluoride diethyl etherate (BF_3_OEt_2_; 42 mL) was added while stirring (600 rpm) at 62 °C under a nitrogen atmosphere. The solution was then stirred under the same conditions for a further 20 h. Subsequently, the supernatant was decanted to obtain a crude product, repeatedly washed with anhydrous methanol, and dried under vacuum at 50 °C to obtain GC [30,31,32].

#### 2.2.3. Synthesis of MSN–COOH–GC

Fifty milligrams of MSN–COOH was added to 25 mL of dimethylformamide (DMF), and 250 mg each of EDC and NHS were then added while stirring and activated at room temperature for 4 h. Another 25 mL of 6 mg/mL GC was added to the activated mixture and reacted at 40 °C for 24 h. After the reaction was complete, the mixture was centrifuged at 12,000 rpm for 10 min, and the product was washed three times with absolute ethanol and dried at 50 °C [45,46,47].

#### 2.2.4. Synthesis of MSN–COOH/GC

Thirty milligrams of MSN–COOH was added to a mixture of 5 mL of PBS (pH 7.4) and 5 mL of GC solution (5 mg/mL), and this mixture reacted at 25 °C for 24 h at a stirring speed of 360 rpm. After the reaction was complete, the mixture was centrifuged at 12,000 rpm for 10 min, and the product was dried in an oven at 50 °C to obtain MSN–COOH/GC.

### 2.3. Preparation of MSN–COOH-Loaded LV

Thirty milligrams of MSN–COOH and 30 mg of LV were added to 10 mL of PBS (pH 7.4) and reacted at 25 °C for 24 h at a stirring speed of 360 rpm under dark conditions. The product processing was the same as in Section 2.2.4. The obtained product is denoted herein as LV@MSN–COOH.

### 2.4. Preparation of MSN–COOH/GC-Loaded LV

Thirty milligrams of MSN–COOH was added to a mixed solution of 5 mL of PBS (pH 7.4) and 5 mL of a GC solution (5 mg/mL), and the mixture was uniformly stirred. Subsequently, 30 mg of LV was added and reacted in a dark environment at 25 °C at a 360 rpm stirring speed for 24 h. The product processing was the same as in Section 2.2.4. The obtained product is denoted herein as LV@MSN–COOH/GC.

### 2.5. Preparation of MSN–COOH–GC-Loaded LV

Twenty-five milligrams of MSN–COOH–GC was added to 10 mL of PBS (pH 7.4), and 25 mg of LV was added under stirring and reacted in a dark environment at 25 °C at a 360 rpm stirring speed for 24 h. The product processing was the same as in Section 2.2.4. The obtained product is denoted herein as LV@MSN–COOH–GC.

### 2.6. MSN–COOH and MSN–COOH/GC Zeta Potential (ς)

The zeta potentials of MSN–COOH and MSN–COOH/GC were measured using a Malvern Zetasizer Nano ZS90 (Malvern Instruments, Worcestershire, UK). MSN–COOH and MSN–COOH/GC were formulated into a 0.1% suspension using PBS (pH 7.4) as a solvent.

### 2.7. Morphology Observations

Transmission electron microscopy (TEM) images were obtained with a JEM-1200EX (JEOL (Beijing) CO., LTD., China) transmission electron microscope operating at 120 kV. The samples were prepared by evaporating a drop of nanoparticulate water solution on an ultrathin carbon-supported copper mesh. Scanning electron microscopy (SEM) micrographs were performed using a SU8020 field-emission scanning electron microscope (HITACHI, Tokyo, Japan) at 20 kV. The samples were not electrically conductive and required gold-spray treatment.

### 2.8. Other Characterizations

Small-angle powder X-ray diffraction (XRD) measurements were carried out on an Empyrean Sharp X-ray Diffractometer (PANalytical B.V., Almelo, The Netherlands) using Cu Kα radiation (λ = 1.54 Å) at 45 kV and 40 mA. Fourier transform infrared (FT-IR) spectra were obtained on an IRAffinity-1 infrared spectrophotometer (Thermo Fisher Scientific, Bar Harbor, ME, USA) in the range of 400–4000 cm^−1^ using the KBr pellet technique. Thermogravimetric analysis (TGA) was carried out on an STA 449 F3 Jupiter (NETZSCH-Gerätebau GmbH, Selb, Germany) from 25 to 900 °C with a heating rate of 5 °C/min under a nitrogen atmosphere. Nitrogen sorption isotherms were measured on a Micromeritics ASAP 2460 (Micromeritics Instrument (Shanghai) Ltd., Shanghai, China) surface area and porosity analyzer at 77 K. The surface area, pore volume, and pore size were calculated using the Brunauer-Emmett-Teller (BET) model and the Barrett-Joyner-Halenda (BJH) method, respectively.

### 2.9. In Vitro Drug Release

We measured the absorbance of different concentrations of LV at 282 nm and 353 nm using an ultraviolet spectrophotometer (Shimadzu UV-2550, Kyoto, Japan) and established a standard curve. Two stock standard solutions (100 µg/mL) were prepared by dissolving 10 mg of LV in 100 mL of PBS (pH 7.4) and hydrochloric acid (pH 1.2). In addition, the stock standard solution was diluted with PBS and hydrochloric acid to yield seven standard solutions containing varying concentrations of LV (5, 10, 15, 20, 25, 30, and 35 µg/mL) to be used in the linearity assessment. We measured the absorbance of the standard solution with an ultraviolet–visible light (UV–Vis) spectrophotometer (Shimadzu UV-2550, Kyoto, Japan) and made a standard curve. The in vitro release was studied using a dialysis bag method. Briefly, LV@MSN–COOH and LV@MSN–COOH/GC were placed in dialysis membrane bags (molecular weight cut-off (MWCO) 8–14 kDa, Solarbio, Beijing, China) and immersed in a fresh dissolution medium of PBS (pH 7.4) or HCl (pH 1.2). The entire system was placed in a shaker incubator (Shanghai Jing Hong Laboratory Instrument Co., Ltd., Shanghai, China) set at 100 rpm at 37 °C. At predetermined time intervals, 5-mL samples were withdrawn and replaced with equal volumes of fresh media to maintain the sink condition. The sample was quantitatively analyzed at 282 nm or 353 nm over a period of 2 h.

### 2.10. Cell Culture

The human colorectal cancer cell line SW620 was purchased from the Type Culture Collection of the Chinese Academy of Sciences (Shanghai, China) and cultured in DMEM supplemented with 10% fetal bovine serum (FBS) and 100 units/mL antibiotics at 37 °C in an humidified incubator with 5% CO_2_.

### 2.11. Determination of Biocompatibility of LV, MSN–COOH, and MSN–COOH/GC

The log phase of SW620 was collected by trypsinization and inoculated into 96-well plates (2 × 10^4^ cells/well). After incubation for 24 h under standard growth conditions, the original culture solution was discarded and a new culture medium of LV, MSN–COOH, and MSN–COOH/GC at concentrations of 50 and 100 µg/mL was added. The mixture continued to incubate for 48 h. Then, 20 μL of 3-(4,5-dimethylthiazol-2-yl)-2,5-diphenyltetrazolium bromide (MTT; 5 mg/mL) was added to each well, and incubation continued for 4 h. The MTT-containing medium was discarded, and 150 μL of dimethyl sulfoxide (DMSO) was added to each well. The optical density (OD) value was read at 490 nm using a Microplate reader (Synergy H1, BioTek, Biotek Winooski, VE, USA).

### 2.12. Cytotoxicity Assay of Combined Action of 5-FU, 5-FU@MSN–NH_2_/GC, and LV@MSN–COOH/GC

Following inoculation and incubation as described above, a new culture medium of LV or LV@MSN–COOH/GC with a concentration of 0.0625, 0.125, 0.25, 0.5, 1.0, or 2.0 µg/mL was added. The mixture continued to incubate for 2 h. After 2 h, a culture medium of 5-FU or 5-FU@MSN–NH_2_/GC with a concentration of 0.625, 1.25, 2.5, 5.0, 10, or 20 µg/mL was added. At the same time, the free 5-FU group and blank group were set up. Thus, the concentration ratio of 5-FU to LV was 10:1 and continued to incubate for 48 h. After 48 h, 20 μL of MTT (5 mg/mL) was added to each well, and incubation continued for 4 h. The MTT-containing medium was discarded and 150 μL of DMSO was added to each well. The OD value was read at 490 nm, and the cell inhibition rates of the 5-FU+LV@MSN–COOH/GC group, the 5-FU@MSN–NH_2_/GC+LV@MSN–COOH/GC group, the 5-FU group, and the 5-FU+LV group were calculated.

### 2.13. Uptake Experiment

#### 2.13.1. Fluorescence Microscopy Cell Uptake

The log phase of SW620 was collected by trypsinization and inoculated in a 12-well plate (3 × 10^4^ cells/well) for 24 h. The original culture solution was discarded, and the MSN–COOH and MSN–COOH/GC labeled by fluorescein isothiocyanate (FITC) with a concentration of 50 µg/mL was added, and incubation continued for 4 h. The culture solution was removed, and the cells were rinsed three times with PBS. Fluorescence microscopy (ECLIPSE 80i, NIKON, Tokyo Metropolitan, Japan) was used to analyze the uptake of samples by SW620 cells. In order to evaluate the competitive effect of galactose on the cellular uptake, 2 mg/mL galactose was added 30 min before adding FITC@MSN–COOH/GC. After incubation for 30 min, the medium containing galactose was aspirated, FITC@MSN–COOH/GC (50 µg/mL) was added to the wells, and the results of fluorescence microscopy were compared with those of MSN–COOH/GC without galactose.

#### 2.13.2. Flow Cytometry Cell Uptake

Following inoculation and incubation of FITC-labeled MSN–COOH and MSN–COOH/GC, as described above, the culture solution was discarded, and the cells were collected by trypsinization, centrifuged at 1000 rpm for 5 min, and suspended in PBS. The uptake rate of the cells was analyzed by flow cytometry (BD FACSCanto II, BD Biosciences, San Jose, CA, USA). To evaluate the effect of different concentrations of galactose on cell uptake and to determine whether this process was concentration-dependent, galactose was added at concentrations of 2 and 6 mg/mL 30 min before the application of FITC@MSN–COOH/GC. After incubation for 30 min, the galactose-containing medium was aspirated, FITC@MSN–COOH/GC (50 µg/mL) was added to the wells, and the results were compared with MSN–COOH/GC without galactose.

### 2.14. Human Thymidylate Synthase (TS) Enzyme-Linked Immunosorbent Assay (ELISA)

The log phase of the SW620 cells was collected by trypsinization and inoculated into six-well plates (7 × 10^5^ cells/well). After incubation for 12 h under standard growth conditions, the original culture solution was discarded, and a new culture medium of LV or LV@MSN–COOH/GC (20.0 µg/mL) was added. Two hours later, 20 µg/mL 5-FU@MSN–NH_2_/GC or free 5-FU was added to ensure a 10:1 ratio of 5-FU or 5-FU@MSN–NH_2_/GC:LV or LV@MSN–COOH/GC. At the same time, a separate blank group, a free 5-FU group, and a 5-FU@MSN–NH_2_/GC group were set. The culture continued to incubate for 48 h, and the culture solution, PBS washing solution, and cell suspension were collected in a 10-mL Eppendorf (EP) tube and centrifuged at 1500 rpm for 5 min. The supernatant was discarded and washed twice with PBS. Dilution with PBS ensured that the concentration of cells in each EP tube was about one million per mL. The cells were placed in a refrigerator for repeated freezing and thawing to destroy the cell structure, and the intracellular components were released. The supernatant was collected by centrifugation at 3000 rpm for 20 min. Subsequently, according to the instructions of the enzyme-linked kit, a standard curve was created using the OD value of the standard at 450 nm of the microplate reader as the abscissa and the concentration as the ordinate, and the concentrations of TS in the SW620 cells of the blank group, the free 5-FU group, the 5-FU@MSN–NH_2_/GC group, the free 5-FU+free LV group, the 5-FU@MSN–NH_2_/GC+free LV group, and the 5-FU@MSN–NH_2_/GC+LV@MSN–COOH/GC group were determined.

### 2.15. Statistical Analysis

All experiments were run in triplicate and the acquired data are expressed as means ± SD. Statistical significance was determined using Student’s *t*-test.

## 3. Results and Discussion

### 3.1. Characterization of Nanocarriers

**Figure 2:**
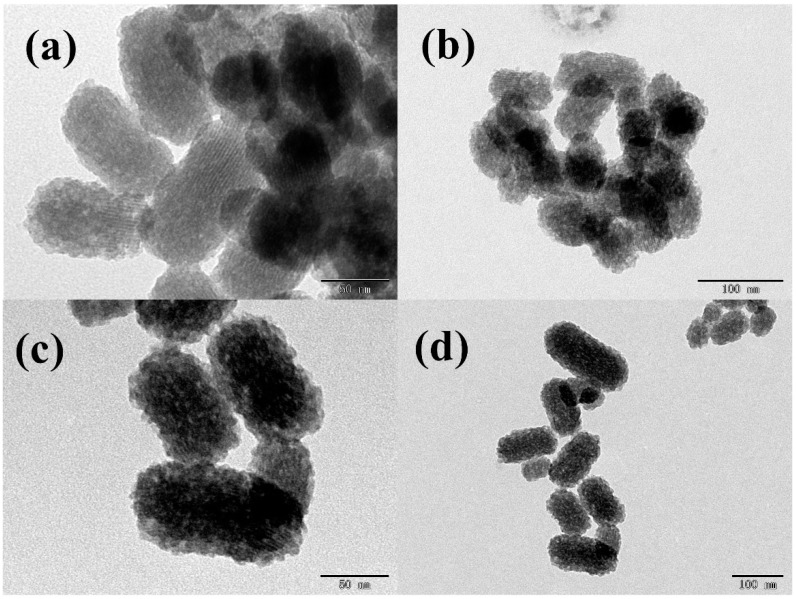
TEM images of MSN–COOH (**a**,**b**) and MSN–COOH/GC (**c**,**d**).

As seen in the TEM image (Figure 2), MSN–COOH (Figure 2a,b) was shown to be a short, rod-like object with a length of 100 nm and a width of 50 nm, with a mesoporous structure inside and a relatively neat arrangement, as is characteristic of Mobil Composition of Matter Number 41 (MCM-41) materials. This indicated the successful synthesis of mesoporous materials. Figure 2c,d show that the structure of MSN–COOH did not change after reacting with GC, and its size remained basically the same, but its surface was covered with a layer of material, indicating the formation of a thin layer of GC.

**Figure 3:**
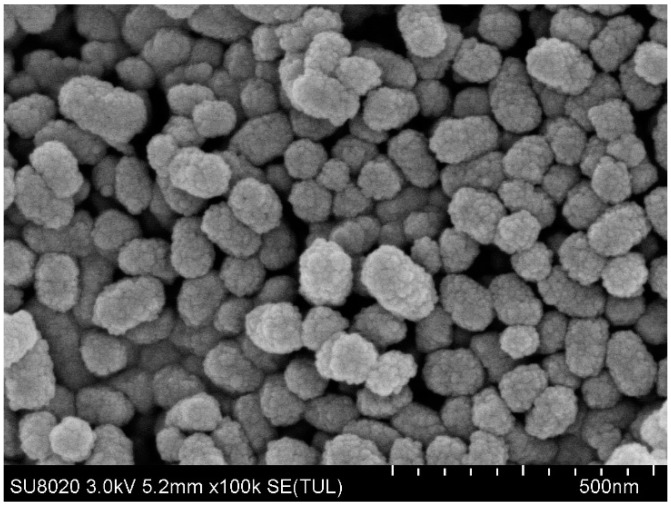
SEM images of MSN–COOH.

**Figure 4:**
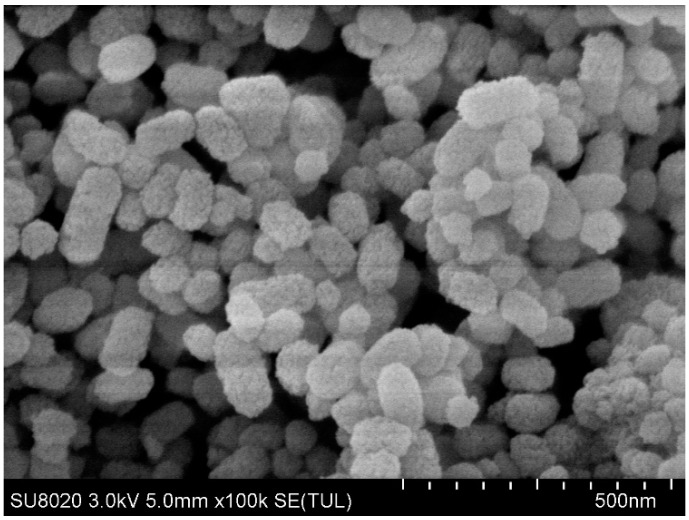
SEM images of MSN–COOH/GC.

The scanning electron microscope results of MSN–COOH and MSN–COOH/GC are shown in Figure 3 and Figure 4, respectively. It can be seen that the structures of MSN–COOH and MSN–COOH/GC are consistent, indicating that MSN–COOH still maintains its nanostructure after GC is added. 

**Figure 5:**
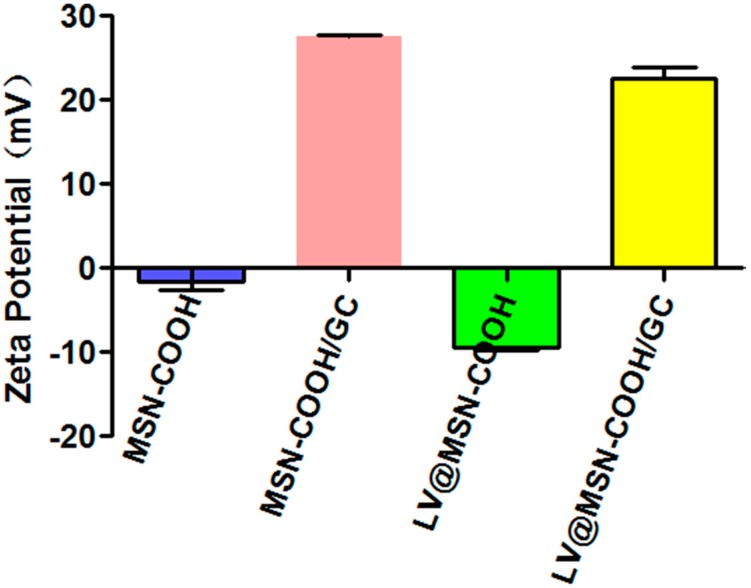
Zeta potential measurements of MSN–COOH, MSN–COOH/GC, LV@MSN–COOH, and LV@MSN–COOH/GC.

The zeta potential results showed a significant difference in surface charge between the different nanocarriers (Figure 5), providing further evidence of the presence of a GC coating on the MSN–COOH. At pH 7.4, the zeta potential of the MSN–COOH nanoparticles was −1.59 mV. After GC treatment, the GC synthesized in this study also retained the C_2_–NH_2_ group for protonation, and its zeta potential increased to ~27.5 mV [30,32], indicating that the GC was successfully coated on MSN–COOH. As shown in Figure 5, with the LV load, the zeta values of MSN–COOH and MSN–COOH/GC decreased to −9.37 mV and 22.56 mV, respectively. From the results, we can see that the zeta potential of MSN–COOH is small and unstable in solution. After adding LV, the LV@MSN–COOH system is also unstable. However, the MSN–COOH and LV@MSN–COOH we prepared were stored in solid form rather than in solution. Moreover, after we added GC, the formed LV@MSN–COOH/GC was stable. At the same time, the results of drug-loaded experiments with different carriers also confirmed that MSN–COOH/GC has a higher drug loading.

**Figure 6:**
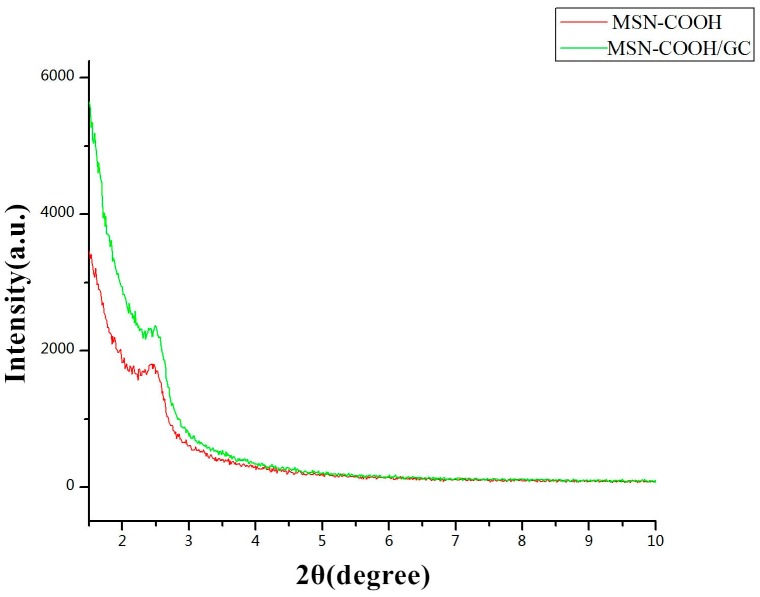
Low-angle powder X-ray diffraction (XRD) of MSN–COOH and MSN–COOH/GC.

Figure 6 indicates the XRD graphs of MSN–COOH and MSN–COOH/GC. The XRD result of MSN–COOH showed a diffraction peak at 2θ values ranging from 2 to 3, indicating it is a mesoporous material [29,48,49]. MSN–COOH did not change its mesoporous structure after reacting with GC, which corresponds with the SEM and TEM results.

**Figure 7:**
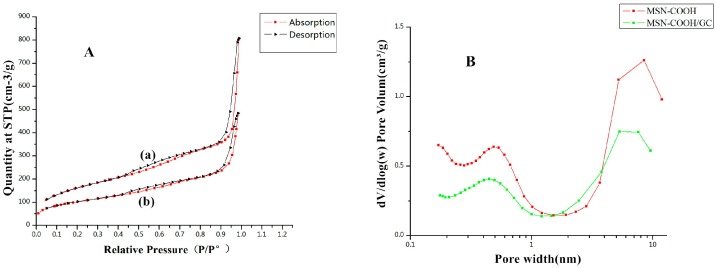
(**A**) Nitrogen adsorption and desorption isotherms for MSN–COOH (a) and MSN–COOH/GC (b). The adsorption branch is shown in black and the desorption branch is shown in red. (**B**) The Barrett-Joyner-Halenda (BJH) pore size distributions of MSN–COOH (a) and MSN–COOH/GC (b).

The surface area and pore size of a mesoporous material play important roles in drug loading and the control of drug release. Figure 7 shows the nitrogen adsorption and desorption of MSN–COOH (Figure 7a) and MSN–COOH/GC (Figure 7b). It can be seen in Figure 7 that the isotherms of MSN–COOH and MSN–COOH/GC are type IV isotherms, in accordance with the International Union of Pure and Applied Chemistry (IUPAC) classification, indicating that they have MSN mesoporous structures, and the coverage of MSN–COOH by GC did not change this structure [29,44]. The surface area of MSN–COOH was 588.49 m^2^/g, while that of MSN–COOH/GC was reduced to 361.88 m^2^/g by GC modification. The pore volume of MSN–COOH was 1.20 cm^3^/g, and that of MSN–COOH/GC was reduced to 0.71 cm^3^/g. From the results, we know that, although our synthesized MSN–COOH and MSN–COOH/GC had a microporous structure, the mesoporous structure was still the main part.

**Figure 8:**
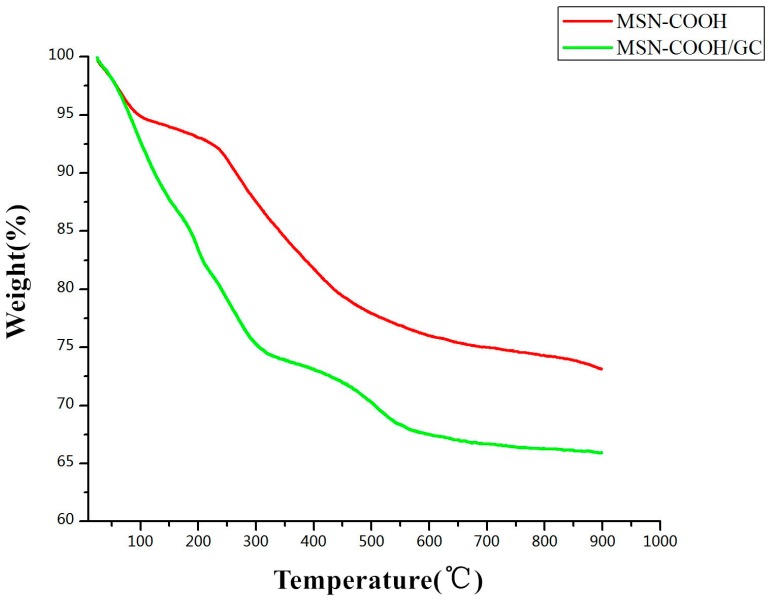
Thermogravimetric analysis (TGA) profiles of MSN–COOH and MSN–COOH/GC.

The results of the thermogravimetric analysis are shown in Figure 8. When the temperature was increased from 25 to 900 °C, MSN–COOH lost 26.86% of its weight in two stages. In the first stage, the weight loss of 6% at 25–150 °C was mainly due to dehydration and dehydroxylation; the weight loss in the second stage was 20.86%, mainly due to the loss of residual groups in the mesopores of MSN–COOH. MSN–COOH/GC lost 34.1% of its weight, and there were also two weight loss stages of 25% and 9.1%, respectively, indicating that a large amount of GC covered the MSN–COOH surface.

**Figure 9:**
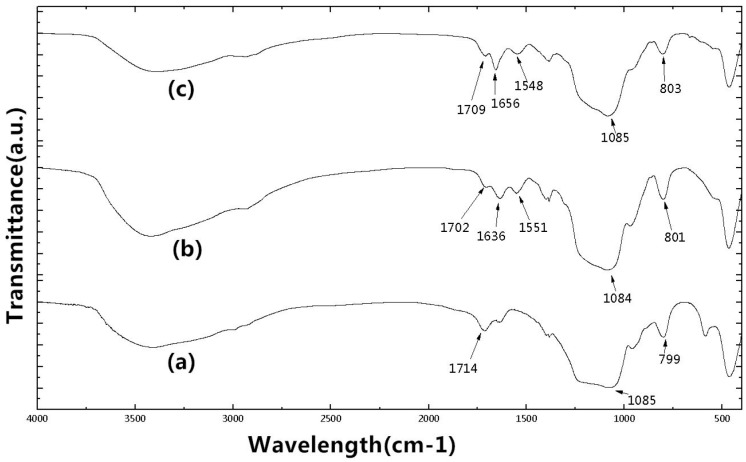
Fourier transform infrared (FT-IR) spectra of MSN–COOH (a), MSN–COOH/GC (b), and MSN–COOH–GC (c).

The FTIR spectra of MSN–COOH, MSN–COOH/GC, and MSN–COOH–GC are shown in Figure 9. Figure 9a shows the infrared result of MSN–COOH. The peak at around 1085 cm^−1^ represents the anti-symmetric stretching vibration absorption peak of the Si–O–Si bond, which showed a symmetric stretching vibration absorption of 799 cm^−1^. There is a peak near 1714 cm^−1^, which represents a carboxyl peak (–COOH), indicating the successful synthesis of MSN–COOH [45,50]. Figure 9b shows the infrared result of MSN–COOH/GC. There is a carboxyl peak near 1702 cm^−1^, and amide I and II band peaks at 1636 cm^−1^ and 1551 cm^−1^, respectively, indicating the presence of GC [31,32]. Figure 9c shows the infrared results of MSN–COOH–GC, where MSN–COOH and GC were combined by a chemical reaction. There is a carboxyl peak at 1709 cm^−1^ and amide I and II band peaks at 1656 cm^−1^ and 1548 cm^−1^, respectively, indicating the presence of GC. The infrared spectra of MSN–COOH, MSN–COOH/GC, and MSN–COOH–GC had no peak at 2250 cm^−1^, which is the stretching vibration absorption peak of cyano (CN), indicating complete hydrolysis of the CN group to a carboxyl group by H_2_SO_4_ [42,44].

**Figure 10:**
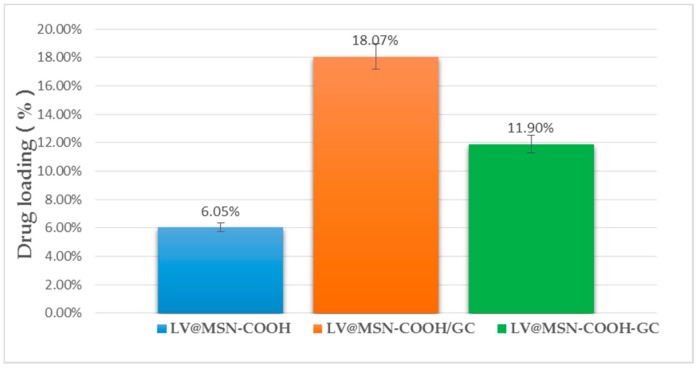
Drug loading of MSN–COOH, MSN–COOH/GC, and MSN–COOH–GC.

Figure 10 shows the LV drug loading of the different carriers. MSN–NH_2_ and MSN–NH_2_/GC were also used in the pre-experiment of this project and their drug loadings were about 6% and 10%; thus, MSN–COOH was selected as the carrier for subsequent experiments. The drug loading of MSN–COOH/GC was shown to be much higher than that of MSN–COOH and MSN–COOH–GC, indicating that GC can increase the drug loading of MSN–COOH.

**Figure 11:**
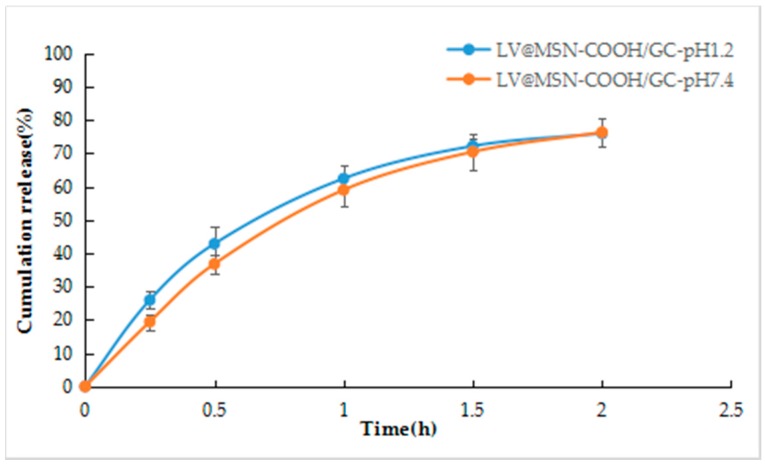
LV release profiles from LV@MSN–COOH/GC.

As shown in Figure 11, LV@MSN–COOH/GC has sustained release at pH 1.2 and pH 7.4. The release was less than 40% at 0.5 h, 80% at 2 h, and 90% at 24 h, which meets the requirements of drug sustained release. 

### 3.2. In Vitro Antitumor Activity of LV@MSN–NH_2_/GC

#### 3.2.1. LV, MSN–COOH, and MSN–COOH/GC Biocompatibility Determination

**Figure 12:**
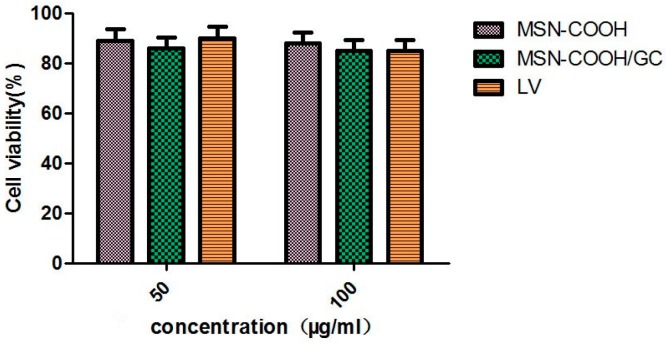
Cell viability assay of LV, MSN–COOH, and MSN–COOH/GC on SW620 cells.

The biocompatibility of LV, MSN–COOH, and MSN–COOH/GC with SW620 cells after 48 h of incubation was detected by MTT assay. The concentrations of LV, MSN–COOH, and MSN–COOH/GC are shown in Figure 12. The cell viability was above 80% when the concentrations were 50 and 100 µg/mL, indicating that these compounds have almost no cytotoxicity and show good biocompatibility.

#### 3.2.2. Cytotoxicity Assay of the Combined Action of 5-FU, 5-FU@MSN–NH_2_/GC, and LV@MSN–COOH/GC

**Figure 13:**
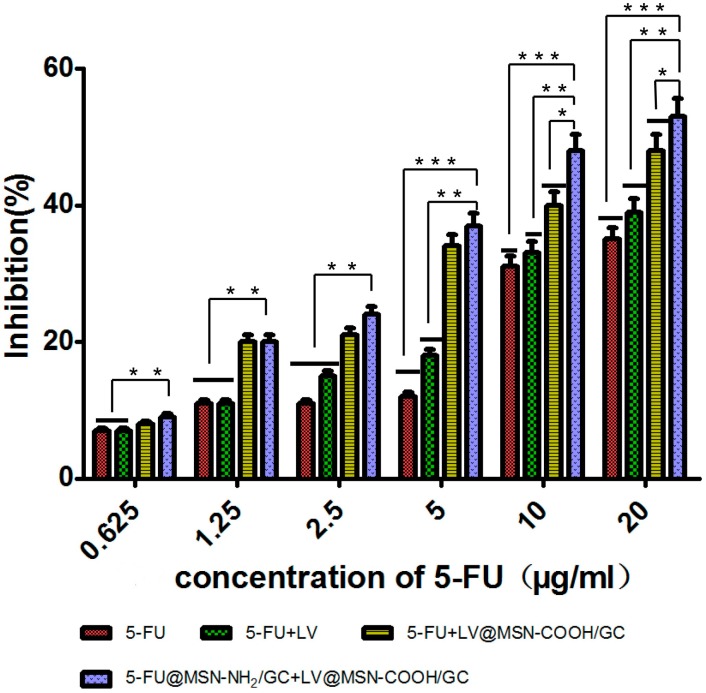
Cell inhibition rates of free 5-fluorouracil (5-FU), 5-FU+LV, 5-FU+LV@MSN–COOH/GC, and 5-FU@MSN–NH_2_/GC+LV@MSN–COOH/GC on SW620 cells at different concentrations. * *p* < 0.05; ** *p* < 0.01; *** *p* < 0.001 (determined with SPSS21).

The cytotoxicities of free 5-FU, 5-FU+LV, 5-FU+LV@MSN–COOH/GC, and 5-FU@MSN–NH_2_/GC+LV@MSN–COOH/GC were detected by MTT assay. The results are shown in Figure 13. Preparations with LV or LV and 5-FU combined were better than pure 5-FU alone. In particular, the inhibition of SW620 was most pronounced when the LV@MSN–COOH/GC preparation was combined with 5-FU@MSN–NH_2_/GC. As a rate-limiting enzyme for DNA synthesis, TS can form a triple complex with FdUMP produced by 5-FU in the presence of folic acid cofactor LV, which competes with the intermediates of the uridine synthesis thymidylate process. It inhibits the formation of deoxythymidylate, which blocks the synthesis of thymidylate and hinders the biosynthesis of DNA, ultimately inhibiting the growth and proliferation of tumor cells. As shown in Figure 13, 5-FU, 5-FU+LV@MSN–COOH/GC, 5-FU@MSN–NH_2_/GC+LV@MSN–COOH/GC, and 5-FU+LV are all concentration-dependent. With an increase in concentration, the inhibitory effects of 5-FU and the combination preparation on SW620 increasingly become stronger. When the concentration of 5-FU was 20 µg/mL, the cell inhibition rates of 5-FU, 5-FU+LV, 5-FU+LV@MSN–COOH/GC, and 5-FU@MSN–NH_2_/GC+LV@MSN–COOH/GC were 35%, 39%, 48%, and 53%, respectively. A *t*-test showed that the rates of 5-FU@MSN–NH_2_/GC+LV@MSN–COOH/GC and 5-FU+LV@MSN–COOH/GC were significantly different (*p* < 0.05), as were those of 5-FU@MSN–NH_2_/GC+LV@MSN–COOH/GC and 5-FU+LV (*p* < 0.01) and 5-FU@MSN–NH_2_/GC+LV@MSN–COOH/GC and 5-FU (*p* < 0.001). The combination of the two nanoagents had a stronger inhibitory effect on SW620 cells, which may be related to the uptake of MSN–NH_2_/GC and MSN–COOH/GC vectors. Specifically, as the uptake increases, more 5-FU and LV enter the cells; thus, the combined effect is more obvious, and the inhibition effect on the cells is stronger.

#### 3.2.3. Uptake Assay

##### Fluorescence Microscopy Cell Uptake

**Figure 14:**
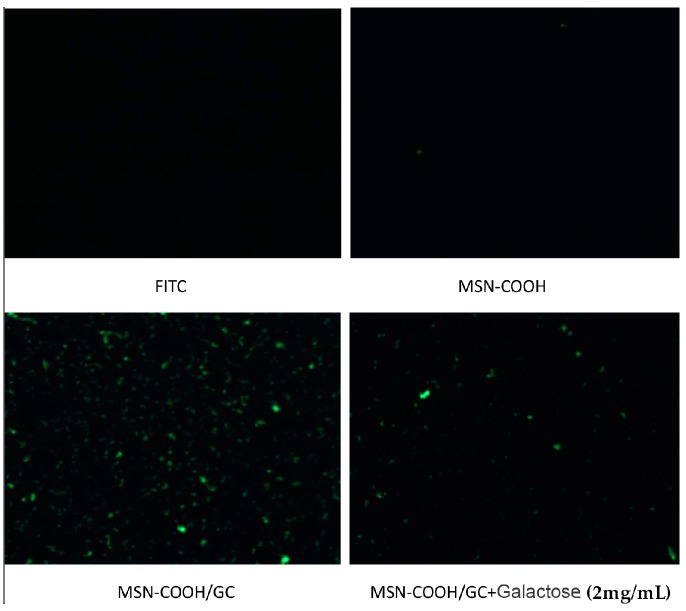
Fluorescence micrographs of SW620 cells internalizing fluorescein isothiocyanate (FITC), FITC@MSN–COOH, FITC@MSN–COOH/GC, and FITC@MSN–COOH/GC+galactose.

The rates of uptake of FITC@MSN–COOH, FITC@MSN–COOH/GC, and FITC@MSN–COOH/GC+galactose (galactose content 2 mg/mL) by SW620 were observed using a fluorescence microscope. It can be seen from Figure 14 that the FITC@MSN–COOH/GC group had a strong fluorescence intensity, and that of the FITC@MSN–COOH/GC group supplemented with 2 mg/mL galactose 30 min earlier was significantly lower, but still stronger than the FITC@MSN–COOH group. Thus, SW620 cells had the highest rate of uptake of MSN–COOH/GC. After adding galactose in advance, the uptake of MSN–COOH/GC by SW620 cells was reduced, and there was no obvious uptake of MSN–COOH. The uptake of MSN–COOH/GC may be related to its association with galactose receptors.

##### Flow Cytometry Cell Uptake

**Figure 15:**
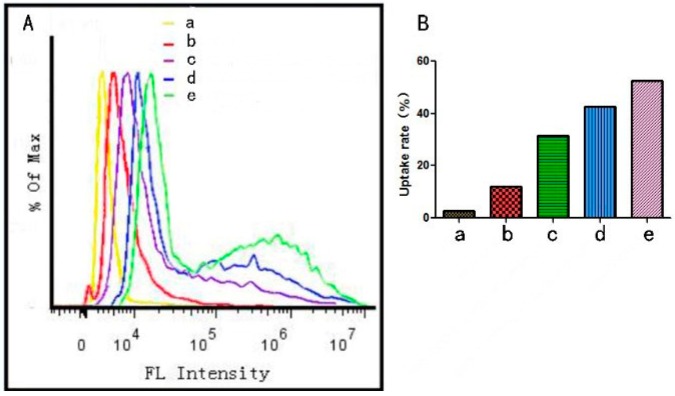
The fluorescence intensity of the samples in SW620 cells (**A**). The uptake rates of the samples in SW620 cells (**B**) (a) FITC, (b) FITC@MSN–COOH, (c) FITC@MSN–COOH/GC+Ga (6 mg/mL), (d) FITC@MSN–COOH/GC+galactose (2 mg/mL), (e) FITC@MSN–COOH/GC.

The uptake rates of FITC, MSN–COOH, MSN–COOH/GC+galactose (6 mg/mL), MSN–COOH/GC+galactose (2 mg/mL), and MSN–COOH/GC were 2.63%, 12.2%, 31.5%, 42.5%, and 52.4%, respectively (Figure 15). The uptake rate of MSN–COOH/GC+galactose (6 mg/mL) was significantly lower than that of MSN–COOH/GC+galactose (2 mg/mL), indicating that the higher the concentration of galactose added to the medium in advance, the faster the galactose binds to the galactose receptor of SW620 cells and the lower binding of MSN–COOH/GC there is to the galactose receptor. The experimental results show that galactose strongly targets SW620 cells.

#### 3.2.4. Human Thymidylate Synthase (TS) Enzyme-Linked Immunosorbent Assay (ELISA)

**Figure 16:**
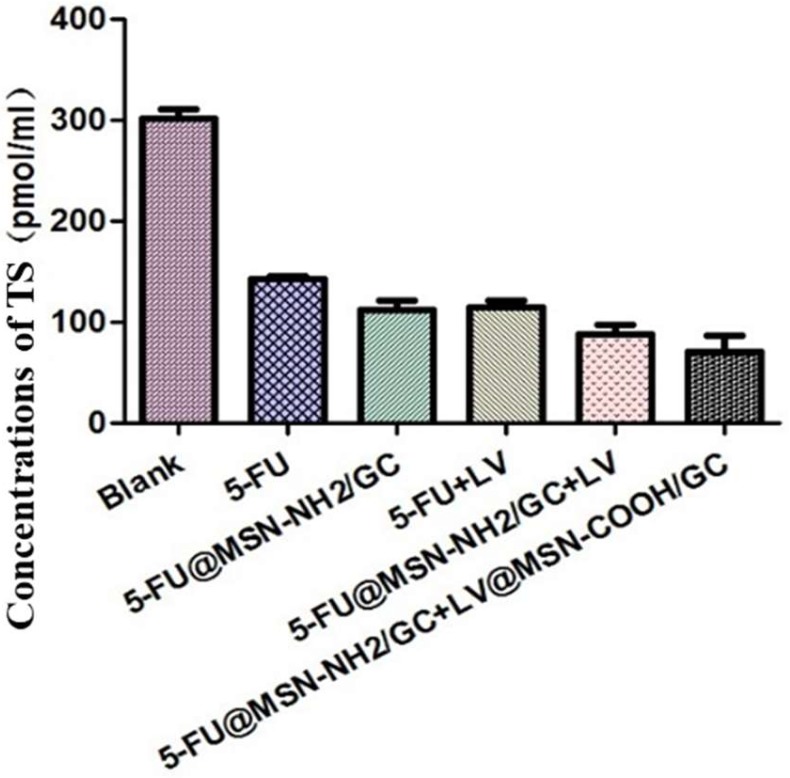
The concentration of thymidylate synthase (TS) in SW620 cells under different experimental groups.

Under the same conditions, the effect of different drugs on the concentration of thymidylate synthase (TS) in SW620 cells was determined. As shown in Figure 16, the concentrations of TS in the blank group, the free 5-FU group, the 5-FU@MSN–NH_2_/GC group, the free 5-FU+free LV group, the 5-FU@MSN–NH_2_/GC+free LV group, and the 5-FU@MSN–NH_2_/GC+LV@MSN–COOH/GC group were 301.29 pmol/L, 142.24 pmol/L, 112.07 pmol/L, 113.79 pmol/L, 87.93 pmol/L, and 69.83 pmol/L, respectively. The concentration of TS in the 5-FU and 5-FU@MSN–NH_2_/GC groups was lower than in the blank group, but it was greater in the 5-FU+LV and 5-FU@MSN–NH_2_/GC+LV groups, indicating that there is combination between 5-FU or 5-FU@MSN–NH_2_/GC and LV. The inhibitory effect on TS increased and its concentration decreased. The concentration of TS in the 5-FU@MSN–NH_2_/GC+LV@MSN–COOH/GC group was lower than in the 5-FU+LV and 5-FU@MSN–NH_2_/GC+LV groups. This showed that the combination between 5-FU@MSN–NH_2_/GC and LV@MSN–COOH/GC is stronger than that between 5-FU and LV, and it is also stronger than that between 5-FU@MSN–NH_2_/GC and LV. Thus, when 5-FU@MSN–NH_2_/GC was combined with LV@MSN–COOH/GC, the inhibitory effect on TS was more obvious, and the effect of inhibiting DNA synthesis in tumor cells was also stronger. The results of this experiment are consistent with the results of the MTT experiment.

## 4. Conclusions

In this study, MSN–COOH was synthesized via a two-step synthesis method. Subsequently, it was coated with galactosylated chitosan (GC), and LV was loaded to construct a nano drug delivery system, which was capable of actively targeting colon cancer cells with high expression of the galectin receptor. A series of characterizations confirmed that the constructed vector had a mesoporous structure and that the GC successfully covered the surface of MSN–COOH. MSN–COOH/GC was shown to have a high LV loading capacity (18.07%). Release studies showed that the release of LV in LV@MSN–COOH/GC is mainly based on diffusion and has a certain level of sustained release. Fluorescence microscopy and flow cytometry confirmed that MSN–COOH/GC can specifically recognize the colon cancer cells (SW620), which highly express the galectin receptor and increase the uptake of MSN–COOH/GC by SW620. The free galactose competition experiment also confirmed the uptake mechanism. In vitro cytotoxicity experiments confirmed that the LV@MSN–COOH/GC constructed in this study and the 5-FU@MSN–NH_2_/GC constructed in our other study have strong combined effects and can enhance the inhibition of SW620 cells. The enzyme-linked assay of thymidylate synthase in SW620 cells also established that the two preparations have a combined effect that enhances the inhibition of thymidylate synthase (TS) in SW620 cells and decreases the concentration of TS in SW620 cells. These results indicate that MSN–COOH/GC, as a promising active targeted delivery vehicle, can actively target the drug to colon cancer cells positively expressing galectin, which has potential value for future applications.
